# Sleep apnea and orofacial pain: an integrative clinical perspective

**DOI:** 10.22514/jofph.2025.046

**Published:** 2025-09-12

**Authors:** Karthikeya Patil, Mahesh Kaggare Puttaraju, Ritu Basavarajappa

**Affiliations:** ^1^Department of Oral Medicine and Radiology, JSS Dental College and Hospital, JSS Academy of Higher Education and Research, 570015 Mysuru, India

**Keywords:** Temporomandibular joint disorders, Obstructive sleep apnea, Bruxism

## Abstract

The intricate interrelationship between obstructive sleep apnea (OSA) and orofacial pain represents a significant clinical challenge that necessitates comprehensive understanding and management. This review elucidates the bidirectional pathophysiological mechanisms underlying these comorbid conditions, wherein OSA demonstrates prevalence rates of 2–9% in adults, with marked gender dimorphism, while orofacial pain conditions affect 10–20% of individuals during their lifetime. The manuscript delineates how sleep-disordered breathing induces compensatory neuromuscular responses, manifesting as increased masticatory muscle activity and nocturnal bruxism, which subsequently precipitates or exacerbates temporomandibular dysfunction (TMD) and associated orofacial pain syndromes. Furthermore, chronic orofacial pain can reciprocally impact sleep architecture, potentially exacerbating sleep-disordered breathing through disrupted jaw mechanics and altered muscle tone. The neurological interface between these conditions involves central nervous system modulation of nociceptive input, contributing to heightened pain sensitivity. Management strategies necessitate a multidisciplinary approach, incorporating continuous positive airway pressure (CPAP) therapy, oral appliances, physical therapy and behavioral interventions, underscoring the importance of concurrent treatment modalities for optimal therapeutic outcomes.

## 1. Introduction

Sleep apnea, particularly obstructive sleep apnea (OSA) and orofacial pain, including temporomandibular disorders (TMD), are commonly encountered clinical conditions that often co-occur but are typically managed in isolation. While these disorders have distinct pathophysiological mechanisms, emerging evidence suggests a significant interplay between them, particularly with respect to their shared anatomical and neurophysiological pathways. The recognition of this intersection has led to growing interest in exploring the bidirectional relationship between sleep apnea and orofacial pain, where one condition may exacerbate or even contribute to the onset of the other. Specifically, the mechanical forces associated with TMD may influence airway dynamics, while the fragmentation of sleep caused by OSA may amplify orofacial pain symptoms, creating a complex clinical picture. The interconnection between sleep apnea, orofacial pain and obesity represents a complex clinical challenge requiring integrated management, with obesity serving as a significant compounding factor through its direct mechanical effects on upper airway patency and orofacial structures, as well as its systemic inflammatory influences on both conditions.

This review seeks to provide a comprehensive, integrative perspective on the relationship between sleep apnea and orofacial pain, with an emphasis on the clinical implications for diagnosis and management. By synthesizing current literature from both sleep medicine and orofacial pain disciplines, the purpose of this manuscript is to inform a more holistic, multidisciplinary approach to treatment. The review aims to highlight how dental professionals, in collaboration with sleep specialists and pain management experts, can enhance the care of patients suffering from these co-existing conditions. Ultimately, this manuscript aspires to contribute to the advancement of clinical practices that address both the functional and symptomatic aspects of sleep apnea and orofacial pain.

## 2. Epidemiology

The epidemiological landscape of sleep-disordered breathing and orofacial pain conditions reveals significant prevalence rates and intricate comorbidity patterns within diverse populations. Obstructive sleep apnea (OSA) demonstrates a notable gender dimorphism, affecting 2–9% of adults, with particularly elevated rates among males (24%) compared to females (9%) in the 30–60 years age bracket, and severe manifestations occurring in 4% and 2% respectively. In pediatric populations, OSA prevalence ranges from 1–4%, predominantly associated with adenotonsillar hypertrophy. Concurrently, orofacial pain conditions exhibit substantial population impact, with 10–20% of individuals experiencing symptoms during their lifetime [1]. Temporomandibular disorders (TMD), a principal etiology of orofacial pain, affects 5–12% of adults, demonstrating female predilection. The intersection of these conditions is particularly noteworthy, with research indicating that 20–50% of OSA patients present with concurrent TMD, suggesting a significant comorbidity pattern potentially mediated through sleep disruption-induced parafunction and increased muscle tension [2].

Recent epidemiological investigations have elucidated a significant association between OSA and TMD. A comprehensive meta-analysis revealed that individuals with TMD exhibit a higher prevalence of OSA, with an odds ratio of 2.61, underscoring the necessity for routine OSA screening in TMD patients [3]. Furthermore, a large-scale cohort study demonstrated that patients diagnosed with OSA have a 2.5-fold increased risk of developing TMD compared to matched controls, highlighting the bidirectional nature of this comorbidity [4]. These findings emphasize the imperative for integrated diagnostic and therapeutic strategies to effectively manage the concomitant occurrence of OSA and TMD.

## 3. Etiology

The etiology of sleep-disordered breathing and orofacial pain conditions encompasses a complex interplay of physiological, anatomical and psychosocial factors. OSA demonstrates a multifactorial pathogenesis, with adipose tissue deposition in the pharyngeal region serving as a primary contributor, particularly in the context of obesity [5]. Anatomical variations, including tonsillar hypertrophy and palatal modifications, significantly influence upper airway patency. Age-related degradation of pharyngeal muscle tone and gender-specific physiological differences contribute to differential risk profiles, with post-menopausal females showing prevalence rates approaching those of males. Genetic predisposition, coupled with environmental factors such as smoking and alcohol consumption, further modulates disease susceptibility.

Orofacial pain conditions, particularly TMD, exhibit equally complex etiological patterns. The pathophysiology encompasses myofascial, articular and neurological components, often manifesting through dysfunction of the temporomandibular joint and associated structures. Nocturnal parafunctional activities, notably bruxism, contribute significantly to muscular hypertonicity and subsequent pain development. Traumatic injuries, whether iatrogenic or accidental, can initiate or exacerbate symptoms, while psychological stressors play a crucial role in symptom manifestation and progression [6].

The interconnection between these conditions is particularly noteworthy, as sleep-disordered breathing often induces compensatory neuromuscular responses, including increased masticatory muscle activity and nocturnal bruxism. This heightened muscle tension, combined with sleep fragmentation-induced stress responses, can precipitate or exacerbate temporomandibular dysfunction and associated orofacial pain syndromes. The resulting hypertonic muscle contractions during respiratory events further contribute to a cycle of pain and dysfunction [7].

## 4. Methodology

The methodology for reviewing the relationship between sleep apnea and orofacial pain from an integrative clinical perspective involves a systematic search of medical literature using PubMed/MEDLINE, Scopus, Web of Science, EMBASE and specialized databases like the Cochrane Library and Dentistry & Oral Sciences Source. The search strategy employs specific terms including “obstructive sleep apnea”, “sleep-disordered breathing”, “orofacial pain”, “temporomandibular disorders”, “bruxism” and “craniofacial pain”, combined with Boolean operators to identify relevant publications from the past twenty years. Studies are screened based on clinical investigations, systematic reviews and meta-analyses that examine bidirectional relationships, shared pathophysiological mechanisms or treatment approaches addressing both conditions. The review prioritizes high-quality evidence including randomized controlled trials, longitudinal studies and practice guidelines from dental and sleep medicine specialties, with particular attention to works addressing multidisciplinary management approaches that integrate sleep medicine, dental and pain management perspectives.

### 4.1 Inclusion criteria

● Studies examining the relationship between sleep apnea and orofacial pain

● Clinical investigations, systematic reviews and meta-analyses from the past twenty years

● Research focusing on bidirectional relationships between the conditions

● Studies addressing shared pathophysiological mechanisms

● High-quality evidence including randomized controlled trials and longitudinal studies

● Practice guidelines from dental and sleep medicine specialties

● Works addressing multidisciplinary management approaches

● Publications integrating sleep medicine, dental and pain management perspectives

### 4.2 Exclusion criteria

● Studies published more than twenty years ago

● Research focusing on only one condition (either sleep apnea or orofacial pain in isolation)

● Low-quality evidence or studies with significant methodological limitations

● Case reports without substantial clinical implications

● Publications not addressing the interrelationship between the conditions

● Studies without clear clinical relevance or application

## 5. Discussion

OSA is a disorder marked by recurrent episodes of partial or complete obstruction of the upper airway during sleep. These episodes result in intermittent cessation of airflow, which leads to hypoxia, fragmented sleep and daytime somnolence. The pathophysiology of OSA primarily involves the collapse of the pharyngeal airway, which occurs when the muscles of the upper airway, especially the genioglossus muscle, fail to maintain airway patency during sleep. The collapse is often influenced by anatomical features such as reduced oropharyngeal space, increased adiposity or altered neuromuscular tone.

The etiology of OSA is multifactorial, with obesity being one of the most significant contributing factors, as adiposity in the neck area contributes to the narrowing of the airway. Aging also plays a critical role by reducing the tone and function of pharyngeal muscles, which becomes particularly pronounced in individuals over the age of 50. Moreover, genetic predisposition and craniofacial anomalies, such as a small mandible or large tongue, further exacerbate the likelihood of airway obstruction.

OSA involves a complex interaction between the central nervous system (CNS) and the respiratory system, where repeated upper airway obstructions during sleep lead to intermittent hypoxia and hypercapnia. These changes activate chemoreceptors in the brainstem, triggering arousals to restore breathing, but the brain often fails to maintain upper airway tone during sleep, causing repeated apneas. This disrupted feedback mechanism contributes to sympathetic nervous system activation, leading to increased heart rate and blood pressure. Chronic intermittent hypoxia and sleep fragmentation also cause neuroinflammation and impair brain function, affecting cognitive processes like memory, attention and executive function. Over time, these processes can lead to long-term brain damage and increased risks of cardiovascular and neurodegenerative diseases [8].

The physiological sequelae of OSA extend beyond sleep disruption. The intermittent hypoxemia associated with the disorder induces systemic inflammation, sympathetic nervous system activation and increased oxidative stress, all of which are associated with a higher risk for cardiovascular diseases, stroke and metabolic disorders (*e.g.*, hypertension and diabetes). Notably, OSA’s effects on systemic health also have significant ramifications for orofacial pain, as disturbances in sleep architecture and muscle tone may contribute to conditions like bruxism and TMD, which are common sources of orofacial pain.

The detailed pathophysiological mechanism between sleep apnea and Orofacial pain is explained in Fig. 1 below:

**Fig. 1.** Pathophysiological mechanism showing the relationship between sleep apnea and orofacial pain.

Orofacial pain encompasses a wide range of disorders affecting the face, jaw and mouth. It is most commonly attributed to TMD, bruxism, dental malocclusions, musculoskeletal pain and neuropathic conditions. TMD is a collective term used to describe a group of disorders affecting the (TMJ), associated musculature, and surrounding structures. The exact etiology of temporomandibular disorders is multifactorial, involving a combination of biomechanical, neurological and psychological factors.

Temporomandibular Joint Dysfunction often arises from joint degeneration, internal derangement or inflammation, typically resulting from trauma or prolonged muscle hyperactivity due to bruxism. The condition is also associated with psychosocial factors such as stress, anxiety and depression, which exacerbate muscle tension and contribute to the perpetuation of pain.

Bruxism, or involuntary grinding or clenching of the teeth, is another prevalent cause of orofacial pain. It is considered a parafunctional activity, often occurring during sleep, and is thought to be exacerbated by stress, sleep disturbances and abnormal occlusion. Bruxism is particularly relevant in the context of OSA, as sleep-related bruxism has been found to be more common in individuals with sleep apnea, potentially as a response to nocturnal airway obstruction. The resultant muscle hyperactivity and joint stress contribute to muscle fatigue, headaches and TMD.

The interrelationship between OSA and orofacial pain is complex, involving both direct and indirect mechanisms. The following discusses the principal ways in which these conditions may interact:

a. Bruxism as a mechanism linking sleep apnea and orofacial pain

Bruxism, especially sleep bruxism, has been strongly linked to OSA. One of the proposed mechanisms is the airway obstruction caused by hypoxia and hypercapnia during apneic events, which leads to a reflex increase in muscle activity in the masticatory muscles. This muscle hyperactivity serves as a response to the airway obstruction, but it also contributes to the development of orofacial pain through muscle strain, tooth wear and the development of TMD. Bruxism is a key factor in the etiology of headaches, myofascial pain and joint dysfunction [9].

b. TMD and its impact on sleep

Conversely, TMD can have a significant impact on sleep quality and may exacerbate or even contribute to the development of OSA. Chronic pain from TMD can disrupt normal sleep patterns, leading to fragmented sleep and poor sleep quality. As a result, individuals with TMD may be more likely to develop sleep disturbances, including central or obstructive sleep apnea, as sleep disorders often exacerbate pain sensitivity and muscle dysfunction. The dysfunction in jaw mechanics and muscle tone associated with TMD can further contribute to upper airway obstruction, especially in individuals with predisposing anatomical factors.

c. Role of neurological factors

There is also emerging evidence suggesting that central nervous system dysfunction may play a role in the comorbidity of sleep apnea and orofacial pain. Both conditions involve neuroplastic changes in the central nervous system, which contribute to the perception of pain and muscle hyperactivity. Studies have suggested that the central nervous system may amplify nociceptive input from both the orofacial region and the upper airway, leading to a heightened sensitivity to pain in individuals with OSA and TMD [10].

The interrelationship between orofacial pain and sleep apnea presents significant clinical challenges due to various confounding factors and mechanisms. These include overlapping symptoms that complicate diagnosis, physiological connections such as OSA-induced muscle tension and bruxism, shared risk factors like obesity, and the neurological phenomenon of central sensitization where sleep disruption heightens pain sensitivity. Additional complications arise from medication side effects that may worsen either condition, psychosocial factors creating feedback loops between pain and poor sleep, and structural abnormalities that simultaneously affect both disorders. The position in which a person sleeps can also influence both conditions concurrently [11]. These complexities create substantial limitations in clinical management, including delayed or incomplete diagnosis when clinicians focus on only one condition, difficulty establishing causal relationships between symptoms, treatment interventions that may improve one condition while exacerbating the other, and challenges in coordinating effective multidisciplinary care. The absence of comprehensive clinical guidelines that address both conditions simultaneously often results in fragmented treatment approaches. Consequently, managing patients with comorbid orofacial pain and sleep apnea requires an integrated diagnostic and treatment strategy that considers the multifaceted interactions between these conditions to achieve optimal outcomes.

## 6. Diagnosis and clinical implications

The diagnosis of sleep apnea is typically made through polysomnography or home sleep testing to evaluate the presence of apneas, hypopneas and overall sleep disruption, with particular attention to the Apnea-Hypopnea Index (AHI) and Oxygen Desaturation Index (ODI). The AHI, which quantifies the number of breathing cessations and reductions per hour of sleep, serves as a primary metric for OSA severity classification (mild: 5–15, moderate: 15–30, severe: >30), while the ODI measures the frequency of significant drops in blood oxygen saturation, providing crucial information about intermittent hypoxemia severity. However, in patients with orofacial pain, especially TMD, sleep apnea may be underdiagnosed because symptoms such as daytime fatigue, snoring, and poor sleep quality may be misattributed to the pain or discomfort itself. Additionally, clinical evaluation of TMD, which involves assessing the temporomandibular joint (TMJ), masticatory muscles and bite alignment, may also miss signs of undiagnosed sleep apnea. Screening tools such as the Epworth Sleepiness Scale and Berlin Questionnaire are valuable for identifying patients at risk for OSA, and their use is crucial in patients presenting with orofacial pain.

Further, patients with TMD may also experience bruxism, which is often associated with sleep apnea and can exacerbate muscle pain and dysfunction. This interrelationship becomes more evident when analyzing sleep studies showing elevated AHI values coinciding with increased masticatory muscle activity and when ODI patterns reveal oxygen fluctuations that may trigger autonomic arousal and muscle activation. This can make distinguishing between the pain arising from TMD and the potential airway-related effects of OSA challenging. High-resolution imaging and 3 dimensional airway analysis can assist in evaluating anatomical factors such as airway obstruction, particularly when correlating these findings with polysomnographic data that includes comprehensive AHI and ODI measurements. Consequently, a dual approach of both clinical examination and sleep diagnostics is essential for accurate identification of these co-existing conditions, with special attention to both respiratory indices and orofacial pain patterns during comprehensive assessment.

## 7. Management

Optimal management of comorbid OSA and orofacial pain necessitates a multimodal therapeutic framework predicated on comprehensive phenotyping and individualized intervention protocols. Primary therapeutic modalities encompass positive airway pressure therapy—with meticulous interface selection to mitigate orofacial discomfort—and custom-fabricated mandibular advancement devices with incrementally titrated protrusion to balance airway patency with temporomandibular joint loading. Adjunctive interventions may include myofunctional therapy to enhance oropharyngeal muscle tone, cognitive-behavioral interventions for pain catastrophizing and insomnia, pharmacological management with targeted muscle relaxants or low-dose tricyclic antidepressants for nociceptive modulation, and selective application of botulinum toxin for masticatory muscle hyperactivity. The cornerstone of successful management remains interdisciplinary collaboration between sleep medicine specialists, orofacial pain practitioners, otolaryngologists and behavioral medicine experts, with treatment sequencing strategically orchestrated to address the pathophysiological mechanisms underlying this complex clinical intersection, thereby optimizing functional outcomes while minimizing iatrogenic complications.

## 8. Limitations and strengths of the study

This integrative review of sleep apnea and orofacial pain presents several notable limitations, including methodological heterogeneity across primary studies, a scarcity of high-quality randomized controlled trials examining bidirectional relationships, diagnostic inconsistencies in both conditions, reliance on subjective outcomes, potential language and publication biases, complex multifactorial etiologies complicating direct relationship isolation, insufficient longitudinal data, nascent understanding of neurobiological mechanisms, and clinical heterogeneity challenging treatment recommendation generalizability. Nevertheless, the review makes valuable contributions as the first comprehensive synthesis connecting these conditions, employing a multidisciplinary approach that identifies robust cross-study associations, proposing a novel theoretical framework linking sleep disruption to pain modulation, critically analyzing current diagnostic tools to guide methodological improvements, developing a preliminary evidence-informed treatment algorithm despite acknowledged uncertainties, and systematically documenting knowledge gaps to establish a clear research agenda for this clinically important but understudied area, thereby providing both clinicians and researchers with valuable perspectives despite the inherent limitations of the current evidence base.

## 9. Conclusion

The comorbidity between sleep apnea and orofacial pain, particularly in the form of bruxism and TMD, highlights the intricate interplay between sleep disturbances, neuromuscular dysfunction and pain perception. Addressing both conditions simultaneously is crucial for improving patient outcomes, as the management of one can directly influence the other. Clinicians should be aware of this interrelationship and adopt a comprehensive, multidisciplinary approach to diagnosis and treatment, considering the potential for exacerbation of symptoms.

## References

[1] Wu K, Gan Q, Pi Y, Wu Y, Zou W, Su X, *et al*. Obstructive sleep apnea and structural and functional brain alterations: a brain-wide investigation from clinical association to genetic causality. BMC Medicine. 2025; 23: 42. 10.1186/s12916-025-03876-8 PMC11770961 39865248

[2] Wang YP, Wei HX, Hu YY, Niu YM. Causal relationship between obstructive sleep apnea and temporomandibular disorders: a bidirectional Mendelian randomization analysis. Nature and Science of Sleep. 2024; 16: 1045–1052. 10.2147/NSS.S476277 PMC11284133 39076457

[3] Machado CAO, de Resende CMBM, Stuginski-Barbosa J, Porporatti AL, Carra MC, Michelloti A, *et al*. Association between obstructive sleep apnea and temporomandibular disorders: a meta-analysis. Journal of Oral Rehabilitation. 2024; 51: 2220–2233. 10.1111/joor.13794 39007230

[4] Da-Cas CD, Valesan LF, Nascimento LPD, Denardin ACS, Januzzi E, Fernandes G, *et al*. Risk factors for temporomandibular disorders: a systematic review of cohort studies. Oral Surgery, Oral Medicine, Oral Pathology, and Oral Radiology. 2024; 138: 502–515. 10.1016/j.oooo.2024.06.007 39079850

[5] Ning R, Chen J, Lu Y, Guo J. Obstructive sleep apnea: a follow-up program in its relation to temporomandibular joint disorder, sleep bruxism and orofacial pain. BMC Oral Health. 2023; 23: 578. 10.1186/s12903-023-03264-9 PMC10440039 37598191

[6] Maini K, Dua A. Temporomandibular syndrome. StatPearls Publishing: Treasure Island (FL). 2023. 31869076

[7] Okura M, Kato T, Mashita M, Muraki H, Sugita H, Ohi M, *et al*. Relationships between respiratory and oromotor events differ between motor phenotypes in patients with obstructive sleep apnea. Frontiers in Neurology. 2023; 14: 1150477. 10.3389/fneur.2023.1150477 PMC10071011 37025207

[8] Thomas CL, Vattikuti S, Shaha D, Werner JK, Hansen S, Collen J, *et al*. Central disorders of hypersomnolence: diagnostic discrepancies between military and civilian sleep centers. Journal of Clinical Sleep Medicine. 2022; 18: 2433–2441. 10.5664/jcsm.10144 PMC9516578 35855527

[9] Bartolucci ML, Incerti Parenti S, Bortolotti F, Della Godenza V, Vandi S, Pizza F, *et al*. Sleep bruxism and orofacial pain in patients with sleep disorders: a controlled cohort study. Journal of Clinical Medicine. 2023; 12: 2997. 10.3390/jcm12082997 PMC10142632 37109339

[10] Duo L, Yu X, Hu R, Duan X, Zhou J, Wang K. Sleep disorders in chronic pain and its neurochemical mechanisms: a narrative review. Frontiers in Psychiatry. 2023; 14: 1157790. 10.3389/fpsyt.2023.1157790 PMC10267346 37324825

[11] Kang JH, Kim HJ. Potential role of obstructive sleep apnea on pain sensitization and jaw function in temporomandibular disorder patients. Journal of Korean Medical Science. 2022; 37: e307. 10.3346/jkms.2022.37.e307 PMC9550636 36217573

